# Broad-Range Bacterial Detection and the Analysis of Unexplained Death and Critical Illness

**DOI:** 10.3201/eid0802.010150

**Published:** 2002-02

**Authors:** Simo Nikkari, Fred A. Lopez, Paul W. Lepp, Paul R. Cieslak, Stephen Ladd-Wilson, Douglas Passaro, Richard Danila, David A. Relman

**Affiliations:** * Stanford University School of Medicine, Stanford, California, USA; †VA Palo Alto Health Care System, Palo Alto, California, USA; ‡Oregon Health Division, Department of Human Services, Portland, Oregon, USA; §California Emerging Infections Program, Berkeley, California, USA; and ¶Minnesota Department of Health, Minneapolis, Minnesota, USA

**Keywords:** DNA, ribosomal, *Neisseria meningitidis*, *Streptococcus pneumoniae*, rRNA, PCR, diagnosis

## Abstract

Broad-range rDNA polymerase chain reaction (PCR) provides an alternative, cultivation-independent approach for identifying pathogens. In 1995, the Centers for Disease Control and Prevention initiated population-based surveillance for unexplained life-threatening infections (Unexplained Death and Critical Illness Project [UNEX]). To address the causes of UNEX cases, we examined 59 specimens from 46 cases by using broad-range bacterial 16S rDNA PCR and phylogenetic analysis of amplified sequences. Specimens from eight cases yielded sequences from *Neisseria meningitidis* (cerebrospinal fluid from two patients with meningitis), *Streptococcus pneumoniae* (cerebrospinal fluid from one patient with meningitis and pleural fluid from two patients with pneumonia), or *Stenotrophomonas maltophilia* (bone marrow aspirate from one patient with pneumonia). *Streptococcus pneumoniae* rDNA sequence microheterogeneity was found in one pleural fluid specimen, suggesting the presence of multiple strains. In conclusion, known bacterial pathogens cause some critical illnesses and deaths that fail to be explained with traditional diagnostic methods.

In 1995, the Centers for Disease Control and Prevention (CDC) initiated a large-scale, population-based surveillance study to detect life-threatening infectious diseases in previously healthy persons 1 to 49 years of age; this study is known as the Unexplained Death and Critical Illness Project (UNEX) (*1*). Surveillance was performed through the CDC Emerging Infections Program sites in California (Alameda, Contra Costa, and San Francisco counties), Connecticut (New Haven County), Minnesota, and Oregon. At the start of the project, the surveillance population base was 7.7 million. The study design was a prospective case series obtained by enhanced passive surveillance. Only cases from which cultures and routine serologic tests failed to provide a microbiologic diagnosis were selected for further study. Work-up of cases was syndrome based and algorithm directed. Case investigation included laboratory analysis by broad-range 16S rDNA polymerase chain reaction (PCR) to identify the presence of bacteria in specimens collected from normally sterile anatomic sites.

Broad-range bacterial PCR is based on the use of primers that recognize conserved sequences of bacterial chromosomal genes encoding ribosomal RNA (rDNA). The resulting amplified rDNA sequences also contain variable regions and provide a reliable basis for the analysis of phylogenetic relationships among different forms of cellular life. Broad-range bacterial PCR has been used to identify previously uncharacterized as well as known bacterial pathogens directly in clinical specimens, including cerebrospinal fluid (CSF), synovial fluid, blood, and heart valve tissue (*2*–*9*). The use of broad-range bacterial 16S rDNA PCR for identifying bacterial culture isolates in the clinical laboratory is well established (*10*), and this technique is useful as a routine supplemental method for direct bacterial detection from clinical specimens (*11*). However, broad-range bacterial rDNA PCR has not previously been applied in a systematic manner for the diagnosis of unexplained deaths and life-threatening illnesses with features suggesting infection. In our study, we examined this issue.

## Materials and Methods

### Patients and Specimens

Patients were recruited as part of the CDC Emerging Infections Program’s UNEX study and met all project inclusion criteria, including absence of important preexisting medical conditions, development of an illness with defined features suggestive of infectious etiology, severity leading to admission to an intensive care unit or to death, and failure of cultivation and routinely available serologic tests to provide a microbiologic diagnosis (*1*). Of specimens available from more than 139 cases fulfilling these criteria, only those from anatomic sites that are usually culture negative in healthy persons and those from patients with syndromes of suspected bacterial etiology were selected for broad-range bacterial PCR analysis. These 59 specimens from 46 patients were collected from July 1995 to January 2000. The median age of the 46 patients (21 female, 25 male) was 18.5 years (range 1 to 47 years). Most had neurologic (n=17) or respiratory manifestations (n=14). Five had cardiac, one had abdominal, and nine had multiorgan manifestations. The patients resided in New Haven County (CT) (n=5), Minnesota (n=6), Oregon (n=20), or three counties in the San Francisco Bay Area (n=15).

### Bacterial Strains

Partial 16S rDNA sequences were determined from three *Streptococcus pneumoniae* clinical isolates obtained from patients in the San Francisco Bay Area during 1997 and 1998 (strains SF10014, SF10175, and SF10314; California Department of Health Services, Berkeley, CA). *Escherichia coli* strain B DNA (Sigma, St. Louis, MO) was used as a positive control and as template for measurement of PCR assay sensitivity in reactions with primers fD1mod and 16S1RR-B (see below).

### Specimen Processing

Specimens were processed in parallel with interspersed negative control specimens. DNA was extracted from serum (n=4), blood (n=25), blood culture (n=9), and bone marrow (n=2) specimens by using the chaotropic properties of guanidine isothiocyanate, followed by DNA extraction and purification by alcohol precipitation with the IsoQuick Nucleic Acid Extraction Kit (ORCA Research, Bothell, WA). Pleural fluid (n=3) was digested with proteinase K (Sigma) and non-ionic detergent, essentially as described (*12*). CSF (n=16) was processed by either of these methods.

### Broad-Range Bacterial rDNA PCR

All specimens were analyzed with PCR methods using at least one of the following four bacterial broad-range 16S rDNA primer pairs: fD1mod (5´-AGAGTTTGATCYTGGYTYAG-3´, corresponding to positions 8-27 in the *E. coli* 16S rRNA gene) (*4*) and 16S1RR-B (5´-CTTTACGCCCARTRAWTCCG-3´, 575-556) (*6*); 63F (5´-CAGGCCTAACACATGCAAGTC-3´) (*13*) and 16S1RR-B; 8F2 (5´-TGGAGAGTTTGATCCTGGCTCAG-3´, 5-27) and 806R (5´-GGACTACCAGGGTATCTAAT-3´, 806-787) (*3*); and 515F (5´-GTGCCAGCAGCCGCGGTAA-3´, 515-533) (*3*) and 13R (5´-AGGCCCGGGAACGTATTCAC-3´, 1390-1371) (*3*). The presence of amplifiable DNA and PCR inhibitors was assessed with human beta-globin gene PCRs and primers PCO4 and GH20 (*3*).

Forty-three specimens from 34 patients were analyzed with primer pair fD1mod/16S1RR-B. Reactions based on these primers contained 10 mM Tris-HCl pH 8.3, 50 mM KCl, 1.5 mM MgCl_2_, 200 µM each deoxynucleoside triphosphates, 2.5 U AmpliTaq LD DNA polymerase (PE Biosystems, Foster City, CA), and 5-µL template, as well as 20 pmol of each primer in a total volume of 50 µL. After a denaturation step of 3 min at 94°C, PCR steps at 94°C for 30 sec, 56°C for 30 sec, and 72°C for 30 sec were repeated 30 to 36 times, followed by an elongation step at 72°C for 7 min in a GeneAmp PCR system 2400 thermal cycler (PE Biosystems). Products were detected by agarose gel electrophoresis and DNA staining with ethidium bromide. In most cases, the presence of product was also assessed by attempting to generate recombinant plasmid clones (see below). PCR amplification conditions used with some other bacterial broad-range primer pairs included 100-µL reaction volumes and a 4-min initial denaturation step. Reactions with all reagents necessary for PCR, including water processed in parallel with clinical specimens, were performed as negative controls.

### DNA Sequence Determination and Phylogenetic Analysis

PCR products were sequenced directly, as well as after cloning, by using the TA or TOPO cloning systems (Invitrogen, Carlsbad, CA). Automated ABI PRISM 373 or 377 DNA sequencers (PE Biosystems) and BigDye Terminator Cycle sequencing chemistry were used for determining DNA sequences. Both DNA strands were analyzed, and base-editing was performed together with manual review of the electropherograms, using AutoAssembler (PE Biosystems). The 16S rDNA sequences were compared with those in the GenBank database by using the BLAST search tool (*14*). In addition, the automated 16S rRNA sequence alignment tool and phylogenetic algorithms from the ARB software package (Technical University of Munich, Germany) were used (*15*). Complete sequences of the four rRNA operons of a *S. pneumoniae* serotype 4 strain were obtained from the Institute for Genomic Research, Rockville, MD (*16*). Unpublished sequence data from the rRNA operons of a different *S. pneumoniae* strain were obtained from SmithKline Beecham, Philadelphia, PA.

## Results

Bacterial 16S rDNA sequences were detected in specimens from 8 of the 46 patients. When the same PCR conditions were used, negative control reactions failed to yield a visible product of the expected size. The absence of large amounts of inhibitors of the PCR reaction in specimens from the remaining 38 patients was demonstrated by the ability to amplify human beta-globin sequences from those specimens. The specimen types with positive bacterial 16S rDNA PCR results were CSF (three patients), pleural fluid (two patients), bone marrow aspirate (one patient), and blood culture bottle material (two patients) (Table 1). The sensitivity of this PCR assay with primers fD1mod/16S1RR-B was 5-50 *E. coli* 16S rDNA gene copies, as assessed by using purified *E. coli* DNA as template.

**Table 1:** Characteristics of the bacterial 16S rDNA broad-range polymerase chain reaction (PCR)-positive cases

Case ID	Sex	Age (yrs)	Duration of antibiotic therapy before specimen obtained	Clinical syndrome(s)^a^	16S rDNA PCR and sequencing results	Specimen	Outcome
XOR6	M	18	10 min	Neurologic	*Neisseria meningitidis*	CSF	Survived
XOR34	M	13	3 days	Neurologic	*N. meningitidis*	CSF	Survived
XCA73	F	19	1 day^b^	Respiratory & neurologic	*Streptococcus* *pneumoniae*	CSF	Survived
XEB44	F	10	3 days	Respiratory	*S. pneumoniae*	Pleural fluid	Survived
XMN22	M	43	2 weeks	Respiratory	*S. pneumoniae*	Pleural fluid	Survived
XOR63	M	29	1 month	Respiratory	*Stenotrophomonas* *maltophilia*	Bone marrow aspirate	Died (no autopsy)
XOR56	M	11	none	Multisystem	*Staphylococcus* *epidermidis*	Blood culture material	Survived
XCT29	F	10	1 week	Cardiac	*Bacillus* sp., *Halomonas* sp., *Enterococcus* sp.	Blood culture material	Survived

^a^The primary clinical syndrome(s) during hospitalization. ^b^A second cerebrospinal (CSF) sample obtained 5 days later also contained *S. pneumoniae* rDNA.

CSF was studied from 14 project cases with unexplained meningitis. Specimens from five subjects were analyzed with broad-range bacterial rDNA primer pairs 8F2/806R and 515F/13R. PCR products of approximately the anticipated size (804 and 876 bp, respectively) were generated from specimens from cases XOR34 and XOR06. From case XOR34, a 1342-bp 16S rRNA gene consensus sequence was generated from the overlapping gene fragments. This sequence shared 99.3% similarity (1332/1342 nucleotide positions) with the four identical 16S rRNA gene sequences from each of the recently published complete *N. meningitidis* serogroup A and B genomes (GenBank accession numbers AL162758 and AE002551); these GenBank sequences were the closest match to the XOR34 case sequence. Direct sequencing of PCR products from case XOR06 generated 654 bp of sequence homologous to the *N. meningitidis* serogroup A and B 16S rRNA genes. Limited CSF from this case prevented a more complete sequence analysis of the bacterial rDNA in this specimen.

CSF from nine other patients was analyzed by using primers fD1mod/16S1RR-B. Two CSF specimens drawn on different days from case XCA73 each generated PCR products whose directly determined sequences were identical (526/526 bp) to the published 16S rDNA sequence from a *S. pneumoniae* reference strain (NCTC 7465T, AJ001246) and with the 16S rDNA sequences from three *S. pneumoniae* strains isolated from patients in the same region and from the same time period (SF10014, SF10175 and SF10314); these reference sequences were determined as part of this study (Figure). The sequences of two clones from each of the amplified XCA73 products were identical to the sequences obtained directly from the PCR products. The CSF specimens from cases XOR06, XOR34, and XCA73 had been collected after empiric antibiotic therapy had begun.

**Figure:**
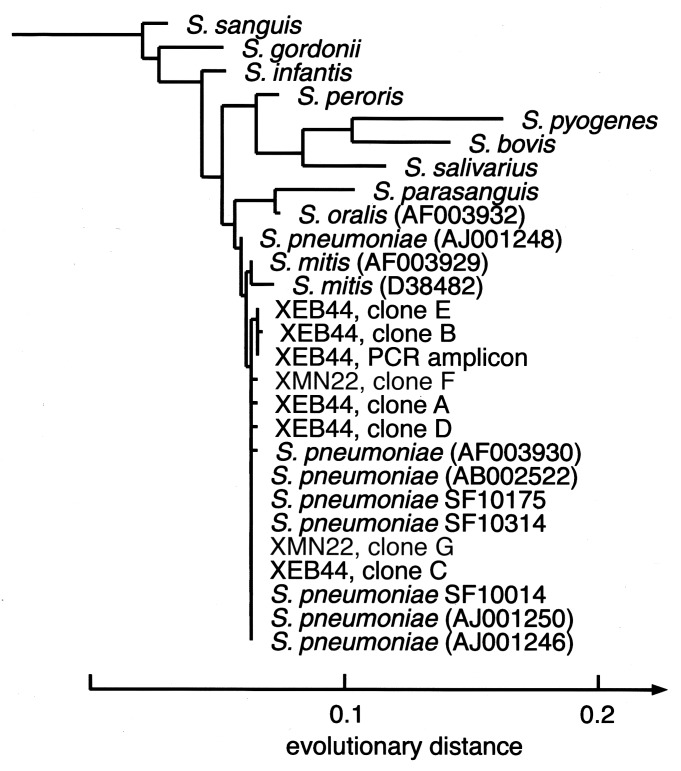
Phylogenetic analysis of the bacterial 16S rDNA sequences obtained from cases XEB44 and XMN22. The tree was rooted with *Staphylococcus aureus* and *Escherichia coli* as outgroups and constructed with a maximum-likelihood algorithm using 468 homologous sequence positions that were selected from a sequence dataset of 497 total positions. *Streptococcus pneumoniae* clinical isolates sequenced for this study are marked as SF10175, SF10314, and SF10014. GenBank database accession numbers for published sequences are given in parentheses. All six sequences from the case XCA73 cerebrospinal fluid were identical to those of the *S. pneumoniae* reference strain (accession no. AJ001246). PCR = polymerase chain reaction.

From two of the three culture-negative pleural fluid specimens (cases XEB44 and XMN22) DNA products of the expected size (~568 bp) were amplified with bacterial broad range primers fD1mod/16S1RR-B. In addition to analysis of cloned molecules from these products, the PCR product from case XEB44 was sequenced directly; insufficient product was obtained from the pleural fluid of case XMN22 for direct sequencing. From the XEB44 pleural fluid specimen, DNA was also amplified with bacterial broad-range primers 63F/16S1RR-B. Three recombinant plasmid clones from this product were characterized by DNA sequencing (Table 2). One clone sequence from each of the pleural fluid specimens (clones C and G in Table 2) was identical (497/497 and 509/509 bp) to an *S. pneumoniae* rDNA sequence deposited in GenBank (reference strain NCTC 7465^T^, #AJ001246)(Figure). However, four other clones from XEB44 (clones A, B, D, and E) and one from XMN22 (clone F) contained one or two positions with variant nucleotides. This variability was confirmed in case XEB44 by examining the sequence obtained directly from the PCR product (Table 2, original PCR product). In contrast, ambiguities were not seen in directly sequenced PCR products from the three clinical isolates (SF10014, SF10175, SF10314), nor in the sequences obtained from case XCA73 CSF.

**Table 2:** Variability in the amplified *Streptococcus pneumoniae* 16S rDNA sequences^a^

	Pleural fluid								Cerebrospinal fluid									
																			XEB44^b^					XMN22			XCA73^c^						Bacterial isolates			Ref. strain^d^
	Specimen 1- PCR analysis 1			Specimen 1-PCR analysis 2		Specimen 1-PCR analysis			Specimen 1-PCR analysis 1			Specimen 2-PCR analysis 1																								
															Corresponding *E. coli* 16S rDNA position	Original PCR product	Clones from PCR products		Clones from PCR products		Clones from PCR products			Original PCR product	Clones from PCR products		Original PCR product	Clones from PCR products								
																														A	B	C					D	E	F	G		H	I		J	K	SF10014	SF10175	SF10314
120	**A/G**	**G**	**G**	**A**	**A**	**A**	**A**	**A**	**A**	**A**	**A**	**A**	**A**	**A**	**A**	**A**	**A**	**A**
**260**	**A/G**	G	**A**	**G**	**G**	**A**	**G**	**G**	**G**	**G**	**G**	**G**	**G**	**G**	**G**	**G**	**G**	**G**
**273**	**A**	**A**	**A**	**A**	**A**	**A**	**G**	**A**	**A**	**A**	**A**	**A**	**A**	**A**	**A**	**A**	**A**	**A**
**279**	**A**	**A**	**A**	**A**	**G**	A	A	A	A	A	A	A	A	A	A	A	A	A
442	T	T	T	T	**C**	T	T	T	T	T	T	T	T	T	T	T	T	T

aNucleotides in bold type differ with the reference strain sequence at the indicated rDNA position. bOne pleural fluid sample was analyzed with PCR on two occasions. cTwo cerebrospinal fluid specimens were taken 5 days apart from the same patient. dPCR = polymerase chain reaction; E. coli = Escherichia coli. eStreptococcus pneumoniae NCTC 7465T (GenBank accession no. AJ001246).

To understand better the variation observed in these pneumococcal sequences, a model of the secondary structure of the *S. pneumoniae* 16S rRNA was constructed, based on the published secondary structure of the *Bacillus subtilis* homologue (*17*) (supplemental data available at http://relman.stanford.edu). Two of the five polymorphic positions are predicted to participate in a “stem” structure and can therefore be assessed for compensatory changes at the paired position. Of the two, only the polymorphism at position 442 creates a noncanonical pairing, thereby suggesting a possible *Taq* polymerase incorporation error. Intrachromosomal allelic variability was another possible explanation for the observed polymorphisms in these pleural fluid *S. pneumoniae* 16S rDNA sequences. However, the complete genome sequence of each of two *S. pneumoniae* strains contains four identical copies of the 16S rRNA operon (*16*) (unpub. data, Michael A. Loretto and Martin Rosenberg). Therefore, our data are consistent with the hypothesis that patient XEB44 was infected in the pleural space by at least two different *S. pneumoniae* strains. Insufficient amounts of data from the patient XMN22 specimen hamper speculation of a similar nature for this case.

PCR analysis of a bone marrow aspirate from a case of fatal respiratory disease (XOR63) showed a 16S rDNA sequence that was 99.8% (527/528 bp) similar to 16S rDNA sequences from *Stenotrophomonas maltophilia* (strain ATCC 13637, #AB008509). This sequence was obtained directly from the PCR product as well as from two recombinant clones; all were identical. A bone marrow aspirate from another patient in our study did not reveal bacterial 16S rDNA sequences.

Two blood culture bottle specimens (from cases XOR56 and XCT29) of nine that were studied gave positive results for bacterial rDNA using PCR primer pair fD1mod/16S1RR-B. All these specimens had been collected from inoculated culture bottles that had failed to exhibit signs of bacterial growth during routine incubation. From one of the two positive specimens (case XOR56), sequences obtained directly from the PCR product and from two recombinant clones were identical (at 535 positions) to the 16S rDNA sequence of a *Staphylococcus epidermidis* type strain (ATCC 14990T, #D83363). From the other PCR-positive blood culture specimen (case XCT29), direct sequencing of the PCR products indicated the presence of multiple, different DNA molecules. Six recombinant clones were sequenced. Two clones were 99.8% (545/546 bp) similar to 16S rDNA sequences from *Enterococcus faecium* (strain DSM20477, #AJ276355) and *E. durans* (strain DSM20633, #AJ276354). One clone was 99.8% similar to a sequence from *Bacillus stearothermophilus* (strain IF012550, #AB021196), one was 98.6% similar (509/516) to a sequence from a *Halomonas sp.* (strain ML-028, #AF139994), and one clone with a truncated sequence was 96.4% similar (323/335 bp) to several *Bacillus* species. The clinical relevance of the rDNA findings in the etiology of these two cases remains uncertain.

## Discussion

In this study, we applied a molecular diagnostic method, broad-range bacterial rDNA PCR to 59 specimens from 46 UNEX cases. Positive findings were obtained for eight of the cases. In six of these cases, the organism detected in the specimens is a known cause of the type of syndrome the patient had.

The diagnosis of infectious diseases with molecular data, rather than from cultivation or serology, requires a careful examination of criteria for causation (*18*) and may require new molecular surveys of host microbial ecology. *Streptococcus pneumoniae* and *N. meningitidis* are common causes of bacterial meningitis in the United States (*19*), and *S. pneumoniae* is a well-known cause of pneumonia and empyema. Despite some unconfirmed reports of *S. pneumoniae* DNA in the serum of healthy persons (*20*–*22*), our failure to find other bacterial sequences in the two pleural fluid specimens with broad-range primers leads us to conclude that *S. pneumoniae* was the cause of these two cases of respiratory tract disease. In CSF, DNA from these organisms has been detected only in meningitis cases. The response of project patients to appropriate antibiotics further supports these conclusions. Similarly, *S. maltophilia* was the only bacterial species detected in a privileged anatomic site (bone marrow) in case XOR63. The known association of this organism with lower respiratory tract disease and septicemia strongly suggests that it caused at least some of this patient’s respiratory disease. However, additional specimens were not available for confirmation; in addition, *S. maltophilia* is ubiquitous in nature and, despite our negative controls, may in theory contaminate laboratory reagents. We speculate that it may have been acquired after the patient's admission to the hospital and contributed to the late stages of the patient’s illness.

In the cases of multisystem failure (XOR56) and cardiac disease (myocarditis, XCT29), the role played by the organisms detected in the blood culture specimens in causing disease is much less clear. Although *S. epidermidis* was also cultivated from the blood of case XOR56 by local physicians, it is a well-recognized blood culture contaminant. Determining its clinical relevance requires additional data. *Bacillus* sp. are also occasional blood culture contaminants, and their DNA may contaminate blood culture media (*23*). An *Enterococcus* sp. is the most likely disease-causing agent among those detected in case XCT29. In general, with reliance on molecular methods, conclusions about causation are most reliably drawn after 1) repeated detection of the same organism in separate specimens from the same patient collected only at clinically relevant time points; 2) direct detection of the organism in situ in areas of pathology (*24*); 3) documentation of response to appropriate therapy; and 4) assessment of specific immune responses. Many of the same concerns and goals are applicable to cultivation-based diagnosis. In practice, especially when case investigation occurs after resolution of illness or death, as in this project, the preferred type and amount of specimens are not always available.

Our findings from these “unexplained” cases raise several important questions. Why weren’t these well-known bacterial pathogens detected during the routine care of these patients? Specimens are not always collected from the appropriate anatomic site, at the appropriate time, in a sufficient amount, nor processed in an optimal fashion. In addition, the sensitivity of cultivation and serology is imperfect, even for organisms amenable to these approaches. Most of the project patients had received broad-range antibiotics before specimens were collected for this study.

[In applying broad-range rDNA PCR to clinical specimens, one confronts the problem of 16S rDNA sequence microheterogeneity. Small discrepancies between directly amplified sequences and reference sequences in public databases pose difficulties for the definition of taxon boundaries, and complicate clinical interpretation of laboratory data (*25*,*26*). In our investigation, the finding of *Streptococcus pneumoniae* 16S rDNA sequence microheterogeneity in the pleural fluid of a patient with culture-negative empyema suggests that some cases of pneumococcal disease, and perhaps other bacterial disease, may be caused by multiple concurrent strains of the same species.

Why have we failed to explain most of these project cases? Are some unexplained cases caused by novel or previously unrecognized pathogens? Our experiments addressed only the possibility of a bacterial cause with bacterial domain-specific broad-range PCR primers; current efforts include broad-range primers for various families of viruses, and for fungi and the *Archaea*. Multiple broad-range primer pairs may be necessary for each group (as we describe for the bacteria) to detect unrecognized organisms with polymorphisms in conserved primer recognition sites.

In addition to the specimen problems listed above, PCR inhibitors may cause some false-negative results. The timing of specimen acquisition may also be relevant, since the average delay between onset of illness and specimen collection was 9 days for our PCR-positive cases versus 16 days for all cases studied with broad-range PCR. Although bacterial DNA persists longer than cultivatable organisms after therapy is initiated (*27*), the former still has a limited half-life. Finally, it must be kept in mind that some of these cases may not have a microbial cause.

Applying broad-range bacterial PCR to this set of difficult clinical cases raised several important points about the future use of this diagnostic approach. First, certain specimen types, such as CSF (3/16 specimens positive), pleural fluid (2/3 positive), and bone marrow (1/2 positive), may provide higher diagnostic yield in bacterial disease than other types, such as serum and blood. Second, the microbial sequence “background” in clinical specimens from healthy persons (e.g., blood) must be better characterized before findings from ill persons can be reliably interpreted. Third, sequence microheterogeneity in anatomically isolated sites of microbial disease may be more common than previously assumed. Our findings from the XEB44 pleural fluid illustrate this point. There are several possible explanations for the *S. pneumoniae* 16S rDNA heterogeneity in this specimen (see above); however, we believe that at least some of the heterogeneity is best explained by the presence of two (or more) *S. pneumoniae* strains. To our knowledge this is the first case of invasive disease associated with multiple *S. pneumoniae* strains, although dual infections with other pathogens have been described (*28*,*29*). In fact, carriage of multiple strains of *Haemophilus influenzae* was recently shown to correlate with an increased risk for otitis media in children (*30*), and mixed-strain infections with *Mycobacterium tuberculosis* have also been reported (*31*). Multiple-strain infections with *S. pneumoniae* may have been previously missed with culture-based methods because of the common laboratory practice of subculturing a single representative when there is only one apparent colony morphotype.

The search for microbial causes of unexplained illnesses and deaths must continue to integrate traditional and molecular methods and will inevitably challenge assumptions about the mechanisms of microbial disease causation. Refined techniques, novel approaches, and study of other populations such as immunocompromised or impaired hosts are likely to provide new insights into the spectrum of infectious diseases.
